# A phase I/II study of the combination of panobinostat and carfilzomib in patients with relapsed or relapsed/refractory multiple myeloma: Final analysis of second dose‐expansion cohort

**DOI:** 10.1002/ajh.26088

**Published:** 2021-01-28

**Authors:** Jesus G. Berdeja, Tara K. Gregory, Edward A. Faber, Lowell L. Hart, Joseph R. Mace, Edward R. Arrowsmith, Ian W. Flinn, Jeffrey V. Matous

**Affiliations:** ^1^ Sarah Cannon Research Institute Nashville Tennessee; ^2^ Tennessee Oncology PLLC Nashville Tennessee; ^3^ Colorado Blood Cancer Institute Denver Colorado; ^4^ Oncology Hematology Care Cincinnati Ohio; ^5^ Florida Cancer Specialists Fort Myers Florida; ^6^ Florida Cancer Specialists St. Petersburg Florida; ^7^ Tennessee Oncology PLLC Chattanooga Tennessee

## Abstract

The maximum tolerated dose of the panobinostat and carfilzomib combination in patients with relapsed/refractory multiple myeloma (RRMM) was not reached in our previous dose‐escalation study. We report additional dose levels in the phase I/II, single‐arm, multicenter, standard 3 + 3 dose‐escalation expansion‐cohort study (NCT01496118). Patients with RRMM were treated with panobinostat 30 mg, carfilzomib 20/56 mg/m^2^ (N = 3), or panobinostat 20 mg, carfilzomib 20/56 mg/m^2^ (N = 33). Treatment cycles lasted 28 days; panobinostat: days 1, 3, 5, 15, 17, 19; carfilzomib: days 1, 2, 8, 9, 15, 16. For dose level 6 (DL 6), median age was 63 years (range, 49–91 years), 60.6% were male, 42.4% were high risk. Patients received a median of two prior therapies (range 1–7); proteasome inhibitors (PI; 100%), immunomodulatory imide drugs (IMiD; 78.8%), and stem cell transplant (36.4%); 48.5%, 51.1%, and 24.2% were refractory to prior PI or prior IMiD treatment or both, respectively. Patients completed a median of seven (range 1–40) treatment cycles. Overall response rate (primary endpoint) of evaluable patients in the expansion cohort (N = 32): 84.4%; clinical benefit rate: 90.6%. With a median follow‐up of 26.1 months (range, 0–72.5 months), median (95% CI) progression‐free survival, time‐to‐progression and overall survival of patients was 10.3 (6.1, 13.9), 11.7 (5.6, 14.5), and 44.6 (20.8, N/A) months, respectively. Common adverse events (AEs) included thrombocytopenia (78.8%), nausea (63.6%), fatigue (63.6%), diarrhea (51.5%), and vomiting (51.5%). Seven patients had serious treatment‐related AEs. There was one treatment‐related death. In conclusion, panobinostat plus carfilzomib is an effective steroid‐sparing regimen for RRMM.

## 1 INTRODUCTION

Proteasome inhibitors (PIs) and immunomodulatory imide drugs (IMiDs) have served effectively as the backbone of various multiple myeloma (MM) therapeutic strategies with excellent results.^1^, ^2^, ^3^ Unfortunately, the majority of patients will eventually progress and more treatment options are needed. Panobinostat is a pan‐histone deacetylase inhibitor (HDACi) that affects multiple cellular pathways, has the ability to resensitize refractory MM cells, and has demonstrated synergistic effects with PIs in preclinical studies.^4^, ^5^, ^6^ The MM cells are highly dependent on the proteasome system to degrade proteins^7^; however, chronic exposure to PIs can cause aggresome formation, which contributes to acquired resistance and results in a poor prognosis for patients.^8^ Panobinostat enhances the anti‐MM activity of PIs by inhibiting the aggresome protein degradation pathway via acetylation of proteins involved in multiple oncogenic pathways.^6^ Panobinostat is approved for the treatment of relapsed/refractory MM (RRMM) in combination with bortezomib and dexamethasone in patients who have received ≥2 prior lines of therapy, including bortezomib and an IMiD.^9^ This regimen was approved based on the results of the PANORAMA 1 study sub‐group analysis, which demonstrated improved efficacy in refractory patients.^10^ However, the study dosed bortezomib intravenously (IV), and high incidences of grade 3/4 hematological and gastrointestinal toxicities were reported.^10^ Thus, new treatment combinations of agents with minimal overlapping toxicities are of great interest to reduce the treatment burden for patients. Carfilzomib is a second‐generation PI approved as a single agent with or without dexamethasone, and in combination with lenalidomide and dexamethasone.^11^ Carfilzomib has a toxicity profile different than the first‐generation PI, bortezomib.^12^, ^13^ In particular, carfilzomib induces less peripheral neuropathy (PN) than bortezomib but is associated with an elevated risk of cardiovascular adverse events (AEs), including heart failure, hypertension, ischemia, and arrhythmia, as well as pulmonary, renal, and thromboembolic AEs, such as dyspnea, acute kidney injury, and deep vein thrombosis/pulmonary embolism.^13^, ^14^, ^15^

We previously reported the results from our dose‐escalation and dose‐expansion study of the steroid‐sparing combination of panobinostat and carfilzomib in 44 patients with RRMM.^16^ In this study, the maximum tolerated dose (MTD) was not reached with the four initially planned dose levels. The highest dose level used for the dose‐expansion cohort in the phase II part of the study was panobinostat 30 mg administered three times weekly (TIW), and carfilzomib 20/45 mg/m^2^. However, the panobinostat dose was often reduced, resulting in an average dose delivery of 23.6 mg in patients starting at 30 mg. Since our phase II expansion cohort publication, studies have shown that higher carfilzomib doses are feasible on a twice‐weekly schedule in the RRMM setting.^13^ The phase III ENDEAVOR study led to the approval of carfilzomib at 56 mg/m^2^ given twice weekly with dexamethasone in patients with RRMM.^11^, ^13^ Taking into account these data and our prior experience with panobinostat dose reductions, which suggested that carfilzomib dosing may be optimized by capping the dose of panobinostat at 20 mg TIW, our original study was extended. Here, we report the results of two additional dose levels and a subsequent dose‐expansion cohort.

## 2 METHODS

### 2.1 Study design

The primary study design, enrollment criteria, study procedures, and assessments have been reported previously.^16^ Briefly, this was a single‐arm, open‐label, multicenter phase I/II study of the combination of panobinostat and carfilzomib in patients with RRMM (NCT01496118). In this parallel study, two additional dose levels of the panobinostat and carfilzomib combination were evaluated. A standard 3 + 3 dose escalation study design was used. If the additional dose levels were tolerated, additional patients would then be enrolled into an expansion cohort. The primary efficacy endpoint was the overall response rate (ORR; ≥ partial response [PR]). The primary safety endpoint was tolerability. Secondary endpoints included time‐to‐progression (TTP), progression‐free survival (PFS), and overall survival (OS).

This study was conducted according to the ethical principles of the Declaration of Helsinki, in accordance with the International Conference on Harmonization Guideline for Good Clinical Practice. The protocol was approved by the Institutional Review Boards of participating sites and patients were enrolled following written informed consent.

### 2.2 Patients

As reported previously,^16^ eligible patients had measurable MM, defined by International Myeloma Working Group (IMWG) guidelines,^17^ which had progressed during or after at least one previous bortezomib‐containing treatment regimen. Key inclusion criteria also included an Eastern Cooperative Oncology Group performance status 0–2, absolute neutrophil count ≥1000/μL, platelets ≥70 000/μL, and adequate organ function as measured by serum creatinine (<1.5 upper limit of normal) and liver function tests. Patients with QTc >450 msec on screening electrocardiogram (ECG) were excluded from the study. Patients were excluded if they were currently receiving or had received systemic cancer therapy (chemotherapy, biologic therapy) ≤21 days of initiating study therapy, or radiation or high‐dose steroid therapy ≤7 days of initiating study therapy. Patients were also ineligible if they had previously been treated with HDACis, HSP90 inhibitors, or valproic acid for the treatment of cancer, or if they had >2 grade PN or diarrhea.

### 2.3 Treatment procedures

Two dose‐escalation levels were planned (Table S1), dose level 5 (DL 5), and dose level 6 (DL 6). Dose level 5 was the next dose‐escalation up from our previous study. However, as many patients required a panobinostat dose reduction, in DL 6 the panobinostat dose was de‐escalated to 20 mg. Treatment cycles lasted 28 days. Panobinostat 20 mg was administered orally on days 1, 3, 5, 15, 17, and 19 (one‐week‐on, one‐week‐off), and IV carfilzomib 56 mg/m^2^ was administered over 30 min on days 1, 2, 8, 9, 15, and 16. Treatment continued until progressive disease or intolerable toxicity. No dexamethasone or other steroids were planned as therapeutic or prophylactic treatment. Patients underwent triplicate ECG on cycle 1 day 1, and cycle 1 day 5, before and 3 hours after panobinostat dosing. If no issues with QTc prolongation were identified during cycle 1, a single pre‐dose ECG on day 1 of each subsequent cycle was required.

### 2.4 Assessments

All AEs were assessed according to the National Cancer Institute's Common Terminology for AEs (CTCAE) version 4.0.^18^ Responses were assessed using IMWG Uniform Response Criteria,^17^ except for minimal response, which was defined according to the European Group for Blood and Marrow Transplant criteria.^19^ Note, TTP was defined as the interval between first administration of study treatment and tumor progression or last adequate tumor assessment. So, PFS was defined as the interval between first administration of study treatment and disease progression or last assessment/follow‐up, or death due to any cause. Overall survival was defined as the interval from first study treatment until last assessment/follow‐up, or death.

### 2.5 Statistical analyses

The efficacy analysis included all patients who received one or more dose(s) of both carfilzomib and panobinostat and underwent one or more response assessment. The safety analysis included patients who received at least one dose of study treatment. Note, TTP, PFS, and OS distributions were evaluated using Kaplan–Meier methods. Sample size was based on the historic ORR of 18% for single‐agent carfilzomib treatment of RRMM.^20^ A sample size of 27 achieves 80% power to detect an increase in the ORR to 36% (representing a 100% relative improvement) based on a one‐sided test of proportion at an alpha level of 0.10. To account for potential non‐evaluable patients and adjusted relative to the actual number of patients in the phase I part of the study who are treated at the optimal dose level, the sample size was increased by 10%.

## 3 RESULTS

### 3.1 Patient disposition and baseline characteristics

Between May 9, 2013 and December 8, 2014, 36 patients were enrolled across seven centers. Three patients were enrolled to DL 5. Although this dose level was well tolerated, without dose‐limiting toxicities observed, due to emerging data from the dose level 4 expansion and significant late dose reductions of panobinostat, DL 5 with panobinostat 30 mg was not pursued further and instead DL 6 was evaluated and expanded. Thirty‐three patients were enrolled to DL 6 and the rest of the manuscript will reflect DL 6 patients only.^16^

Overall, in DL 6 cohort (N = 33), patients had a median age of 63 years (range 49–91 years), 60.6% were male, and 42.4% were high risk (FISH 1q amp, or t(4;14), or t(14;16), or 17p del, at diagnosis, pre‐treatment or any other time on study). Baseline characteristics are summarized in Table 1. Patients had received a median of two prior therapies (range 1–7); PI (100%; bortezomib: 93.9%), IMiD (78.8%; lenalidomide: 75.7%; thalidomide: 30.3%), and stem cell transplant (36.4%; Table 1). Approximately half of patients were refractory to prior PI treatment (48.5%) or prior IMiD treatment (51.5%), and 24.2% of patients were refractory to both PIs and IMiDs.

**TABLE 1:** Baseline characteristics at dose level 6

	Dose level 6 (n = 33)
Median age, years (range)	63 (49–91)
Number of patients ≥75 years, n (%)	6 (18.2)
Male, n (%)	20 (60.6)
Pretreatment ECOG status, n (%)	
0	16 (48.5)
1	17 (51.5)
Baseline platelet <100 k/uL, n (%)^a^	4 (12.1)
ISS stages, n (%)	
I	10 (30.3)
II	13 (39.4)
III	6 (18.2)
Missing	4 (12.1)
ISS ≥2, n (%)	19 (57.6)
High‐risk patients^b^	14 (42.4)
Median number of prior therapies, (range)^c^	2 (1–7)
Prior treatment class, n (%)	
Prior PI^d^	33 (100.0)
Prior IMiD^e^	26 (78.8)
Prior stem cell transplants	12 (36.4)
Refractory to prior treatment, n (%)	
Refractory to prior PIs	16 (48.5)
Refractory to prior IMiDs	17 (51.5)
Refractory to either prior IMiDs or PIs	25 (75.8)
Refractory to both prior IMiDs and PIs	8 (24.2)

Abbreviations: ECOG, Eastern Cooperative Oncology Group; FISH, fluorescence in situ hybridization; ISS, International staging system; IMiD, immune modulator therapy; PI, protease inhibitor.

^a^ Four patients had baseline platelets less than 100 k/uL.

^b^ Defined as FISH 1q amp, or t(4;14), or t(14;16), or 17p del at diagnosis, pre‐treatment or any other time on study.

^c^ Eight patients had 4 or more lines of prior treatments.

^d^ Prior PI treatments were: bortezomib 94%, carfilzomib 6% and oprozomib 6%.

^e^ Prior IMiD treatments were: lenalidomide 76%, pomalidomide 9% and thalidomide 30%.

### 3.2 Treatment summary

Thirty‐three patients received DL 6 treatment. There were no dose‐limiting toxicities. Overall, patients completed a median of seven (range 1–40) treatment cycles, and eight patients (25.0%) completed over 12 cycles. A total of 22 patients had a panobinostat dose reduction (one dose reduction n = 22; two dose reductions n = 0) and 15 patients had a carfilzomib dose reduction (one dose reduction n = 10; two dose reductions n = 5). Details of the number and types of AEs leading to dose delays and reductions can be found in Table S2. The average panobinostat dose was 18.71 mg and the average carfilzomib dose was 48.63 mg/m^2^. No patients are still on treatment; 12 patients discontinued due to disease progression, 10 due to toxicity, six due to physician discretion (three due to stem cell or bone marrow transplant), four due to patient request, and one patient died on study (grade 5 respiratory failure). At the time of data analysis (June 2020), median follow up was 26.1 months (range 0–72.5 months). Although this regimen was intended as steroid‐sparing, three patients received dexamethasone 4 or 8 mg as premedication prior to carfilzomib dosing. The remaining patients did not receive any steroids as part of their treatment or as premedication.

### 3.3 Efficacy outcomes

All reported efficacy data below refer to the DL 6 cohort (N = 33). However, one patient in the DL 6 cohort was not evaluable for efficacy outcomes as he or she did not receive a dose of carfilzomib before discontinuing treatment.

**FIGURE 1:**
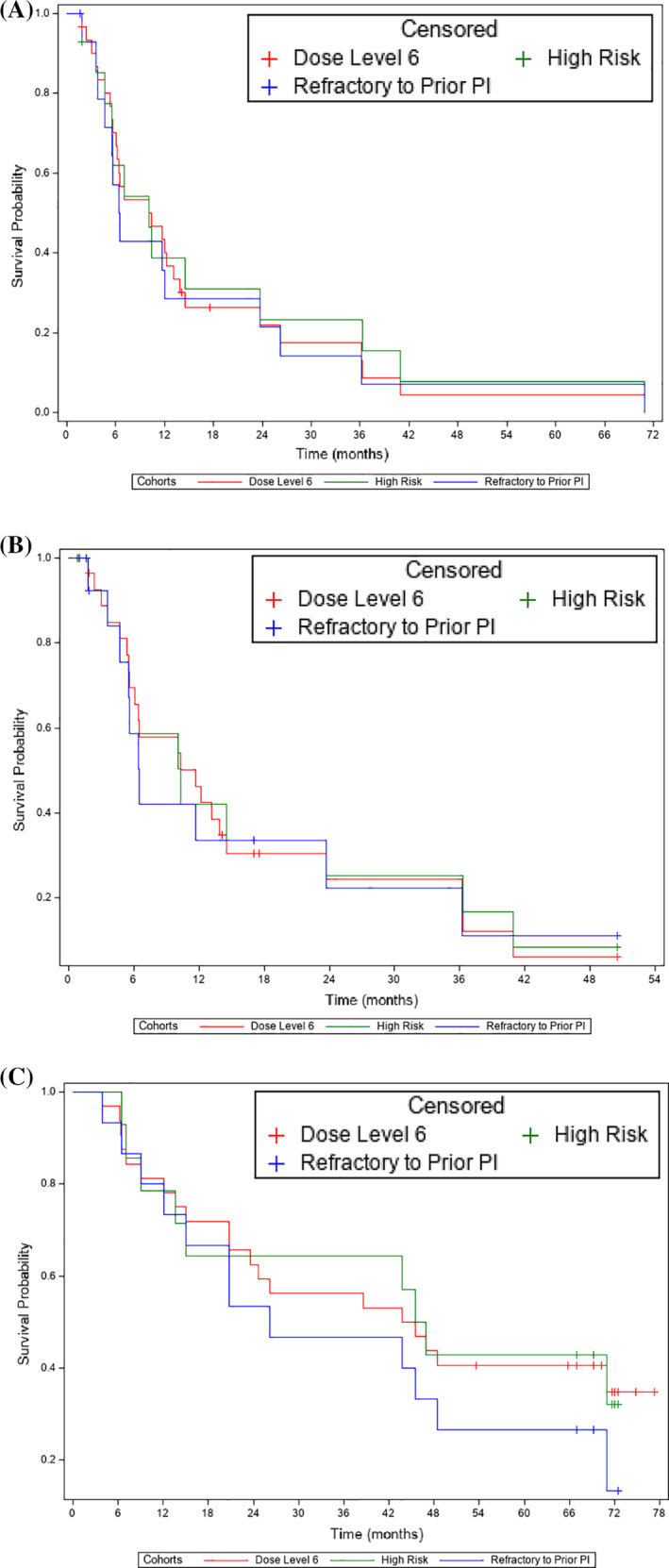
Patient outcomes: (A) PFS; (B) TTP; (C) OS (efficacy analyses), Abbreviations: OS, overall survival; PFS, progression‐free survival; TPP, time‐to‐progression. High risk was defined as FISH 1q amp, or t(4;14), or t(14;16), or 17p del at diagnosis, pre‐treatment or any other time on study

The ORR of all evaluable patients (n = 32) was 84.4% (cycle four: 75.0%; cycle eight: 81.2%; cycle 12: 84.4%) and the clinical benefit rate (CBR, ≥minimum response) was 90.6% (cycle four: 87.5%; cycle 8: 90.6%; cycle 12: 90.6%) (Table 2). The ORR for patients refractory to prior PI (N = 15) was 80.0% and the CBR was 93.3%. And, the ORR for patients refractory to prior IMiDs (N = 16) was 75% and the CBR was 87.5%. Also, ORR for high‐risk patients (N = 14) was 92.9% and the CBR was 100%. Median time to best response was 2 (range, 0.7–22) months. Median (95% CI) PFS was 10.3 (6.1, 13.9) months overall, 6.5 (3.8, 23.7) months in PI‐refractory patients, 6.8 (4.7, 11.7) months in IMiD‐refractory patients, and 10.1 (4.7, 23.7) in high risk patients (Figure 1). Median (95% CI) TTP was 11.7 (5.6, 14.5) months overall, 6.5 (3.6, 23.7) months in PI‐refractory patients, 6.5 (4.7, 11.7) months in IMiD‐refractory patients, and 10.3 (3.6, 36.3) in high‐risk patients (Figure 1). Median (95% CI) OS was 44.6 (20.8, N/A) months overall, 26.2 (9.0, 48.5) months in PI‐refractory patients, 22.2 (7.1, 48.5) months in IMiD‐refractory patients, and 46.2 (9.0, N/A) in high‐risk patients (Figure 1).

**TABLE 2:** Response to treatment (efficacy analyses)

Response assessment	All dose level 6 patients (n = 32)^a^	Not refractory to prior PI (n = 17)	Not refractory to prior IMiD (n = 16)	Refractory to prior PI (n = 15)	Refractory to prior IMiDs (n = 16)	High risk (n = 14)
ORR	27 (84.4%)	15 (88.2%)	15 (93.8%)	12 (80.0%)	12 (75.0%)	13 (92.9%)
CR	2 (6.3%)	1 (5.9%)	0	1 (6.7%)	2 (12.5%)	1 (7.1%)
VGPR and nCR	11 (34.4%)	7 (41.2%)	9 (56.3%)	4 (26.7%)	2 (12.5%)	6 (42.9%)
PR	14 (43.8%)	7 (41.2%)	6 (37.5%)	7 (46.7%)	8 (50.0%)	6 (42.9%)
MR	2 (6.3%)	0	0	2 (13.3%)	2 (12.5%)	1 (7.1%)
SD	3 (9.4%)	2 (11.8%)	1 (6.3%)	1 (6.7%)	2 (12.5%)	0
PD	0	0	0	0	0	0

Abbreviations: CR, complete response; IMiD, immune modulator therapy; MR, minimum response; nCR, near complete response; ORR, overall response rate (CR, VGPR, or PR); PD, progressive disease; PI, protease inhibitor; PR, partial response; SD, stable disease; VGPR, very good partial response.

^a^ One patient was not evaluable as they had one dose of panobinostat and no dose of carfilzomib before discontinuing treatment.

### 3.4 Overall safety outcomes

All patients in DL 6 cohort (N = 33) were evaluable for safety outcomes. Common AEs included thrombocytopenia (78.8%), nausea (63.6%), fatigue (63.6%), diarrhea (51.5%), and vomiting (51.5%). The most commonly reported AEs and all grade ≥ 3 AEs are shown in Table 3. All grade PN and diarrhea were reported by seven (21.2%) and 17 (51.1%) patients, respectively; grade ≥ 3 PN and diarrhea were reported in 3.0% and 9.1% of patients, respectively. Cardiotoxicity was manageable, with most common events being dyspnea in 39.4% and hypertension in 18.2% of patients. More detailed information on cardiotoxicity is presented in Table S3. In total, 39.4% of patients experienced a serious AE, but only seven patients experienced a treatment‐related serious AE; and seven patients had a total of eight treatment‐related serious AEs (pneumonia [two], acute kidney injury, anemia, atrial fibrillation, fever, hemolytic uremic syndrome and thrombocytopenic purpura [one of each]). There was one treatment‐related death; one patient died on study of respiratory failure. Although deemed unrelated to study treatment by the investigator, no alternate cause of death was ever determined and thus is reported as related to study.

**TABLE 3:** Dose level 6 adverse events

AE, n (%)	All grades (1–5)^a^ > 20% of patients (N = 33)	Grades ≥3 > 10% of patients^b^ (N = 33)
Any AE	33 (100)	
Any AE leading to death	1 (3.0)	
Thrombocytopenia	26 (78.8)	20 (60.6)^c^
Nausea	21 (63.6)	2 (6.1)
Fatigue	21 (63.6)	6 (18.2)
Diarrhea	17 (51.5)	3 (9.1)
Vomiting	17 (51.5)	2 (6.1)
Anemia	14 (42.4)	4 (12.1)
Cough	14 (42.4)	
Fever	13 (39.4)	
Dyspnea	13 (39.4)	4 (12.1)
Neutropenia	12 (36.4)	3 (9.1)
Upper respiratory infection	11 (33.3)	
Edema	11 (33.3)	
Headache	11 (33.3)	
Chills	9 (27.3)	
Creatinine levels increased	8 (24.2)	
Anorexia	7 (21.2)	
Dehydration	7 (21.2)	
Dizziness	7 (21.2)	
Arthralgia	7 (21.2)	
Peripheral neuropathy	7 (21.2)	1 (3.0)

Abbreviations: AE, adverse event; CHF, congestive heart failure; SAE, serious adverse event.

^a^ One patient had a serious AE of grade 5 respiratory failure at the beginning of cycle 5 of treatment. One patient experienced a grade 2 “Thromboembolic event” which was deemed unrelated to carfilzomib and panobinostat and no dose modification occurred due to this adverse event.

^b^ Although frequencies of grade 3 or more for diarrhea, nausea, vomiting, neutropenia and peripheral neuropathy were less than 10%, they are provided in the table as they are considered adverse events of special interest.

^c^ 14 (42.4%) patients experienced grade 3 and 6 (18.2%) patients experienced grade 4 thrombocytopenia.

## 4 DISCUSSION

Panobinostat 20 mg administered TIW in a one‐week‐on, one‐week‐off schedule in combination with carfilzomib 20/56 mg/m^2^ was tolerable and effective. The ORR and CBR of the expansion cohort were 84.4% and 90.6%, respectively, and patients had a median (95% CI) PFS, TTP, and OS of 10.3 (6.1, 13.9), 11.7 (5.6, 14.5), and 44.6 (20.8, N/A) months, respectively. Responses were maintained in the subset of patients who were refractory to prior PI or IMiDs but median PFS, TTP, and OS were shorter. Interestingly, this did not apply to patients with high‐risk cytogenetics who had a greater ORR, CBR, and median OS than the overall population, with a generally similar median PFS and TTP. The findings presented here build upon the results of our previous dose‐escalation and dose‐expansion (30 mg panobinostat, 20/45 mg/m^2^ carfilzomib) study.^16^ In our previous study of the combination using lower doses of carfilzomib, the ORR for all patients was 67%, and after a median follow up of 17 months, median PFS was 7.7 months, median TTP was 7.7 months, and the median OS had not been reached.^16^ Although the two studies were initiated at similar times, compared with the initial study,^16^ the patient population in the expansion cohort presented here was not as heavily pretreated (median number of prior therapies: five vs two), but a higher proportion of patients were refractory to their prior treatments (either PI or IMiD: 52% vs 75.8%; both PI and IMiD: 14% vs 24.2%). Due to changes in standard of care, more complex combination regimens are being used as first‐line and second‐line therapy, and maintenance treatment is frequently used in the frontline setting. As such, patients are likely to receive fewer prior lines of therapy before developing refractory disease and requiring novel combinations such as panobinostat and carfilzomib. However, together these data suggest that the panobinostat and carfilzomib combination has the potential to be used before or after the many combination regimens available today, regardless of the number of prior treatment lines.

Although approximately 20% of the population in the current study were over 75 years old, and as such may have been more frail and susceptible to AEs, grade 3/4 AEs were infrequently reported. The most common grade 3/4 AEs in the current study included thrombocytopenia (60.6%), fatigue (18.2%), anemia (12.1%), and dyspnea (12.1%). With the exception of thrombocytopenia, grade 3/4 AE incidences appear to be similar to those we reported previously and in the ENDEAVOR study, and less frequent than those reported with other PI combinations.^16^, ^21^, ^22^ With all the caveats of cross‐trial comparison, the ORR and safety profile of this steroid‐sparing regimen compares favorably to studies of panobinostat and bortezomib regimens. For example, in the phase II PANORAMA 2 study of the bortezomib and panobinostat combination, patients with relapsed and bortezomib‐refractory myeloma (N = 55) and a median of four (range: 2–11) prior lines of therapy achieved an ORR of 34.5% and common grade 3/4 AEs included thrombocytopenia (63.6%), diarrhea (20.0%), and fatigue (20.0%).^21^ Additionally, in the phase III PANORAMA 1 study of the bortezomib, dexamethasone, and panobinostat combination in patients with relapsed (N = 247) or RRMM (N = 134) patients, who had received one (51%), two (32%), or three (17%) prior lines of therapy, achieved an ORR of 60.7%. Common grade 3/4 AEs included thrombocytopenia (67%), lymphopenia (53%), diarrhea (26%), asthenia or fatigue (24%), and PN (18%).^22^

The Food and Drug Administration‐approved panobinostat dosing schema is 20 mg TIW, two‐weeks‐on, one‐week‐off in a 21‐day cycle (as per the PANORAMA studies, as bortezomib is typically dosed in three‐week cycles).^9^ However, differing schemas have been trialed to optimize efficacy and minimize toxicity, particularly potential overlapping toxicities with PIs, as well as to align with the four‐week dosing cycles commonly used for other anti‐myeloma agents. Various dosing schemes of the panobinostat and carfilzomib combination have also been studied by other groups to optimize efficacy and minimize toxicity. In patients who had received a median of four (1–8) prior lines of therapy, Kaufman et al., determined the MTD to be carfilzomib 36 mg/m^2^ and panobinostat 20 mg TIW (3‐weeks‐on, 1‐week‐off, every 28 days).^23^ This dosing schedule resulted in an ORR and CBR of 63% and 68%, respectively, with a median PFS and OS of 8 and 23 months, respectively. The most common grade 3/4, treatment‐related AEs were thrombocytopenia (41%), fatigue (17%), and nausea/vomiting (12%).^23^ Manasanch et al., also studied the panobinostat and carfilzomib combination with dexamethasone.^24^ Patients who had received a median of four (2–16) prior lines of therapy were treated with panobinostat 20 mg TIW (two‐weeks‐on, two‐weeks‐off, every 28 days) and carfilzomib 20/45 mg/m^2^. Dexamethasone 40 mg weekly could be added at the investigator's discretion and 4 mg weekly could be used in patients intolerant to steroids. Interestingly, Manasanch et al. found that adding dexamethasone to the regimen improved the ORR from 18% to 53%, CBR from 36% to 65%, and prolonged OS from 10.1 to 18.2 months. However, grade 3/4 thrombocytopenia was reported in 64% of patients.^24^ The steroid‐sparing combination of panobinostat and carfilzomib is feasible and efficacious and would be an excellent option for the subset of patients intolerant of steroids. However, in patients who can tolerate steroids, could the addition of dexamethasone improve on these results? The ENDEAVOR study reported an ORR 77% and PFS of 18.7 months with the combination of carfilzomib 56 mg/m^2^ twice weekly and dexamethasone.^13^ Our current study showed a comparable ORR of 84%, albeit with an inferior PFS of 10.3 months. As stated earlier, the incorporation of dexamethasone makes a direct comparison of these two regimens difficult. Furthermore, the patient population differed significantly from a standpoint of prior PI use. It is notable that the percentage of patients with prior exposure to bortezomib was much higher in our current study (94% vs 54%) as was the percentage of patients refractory to bortezomib (49% vs 0%). This could account for some of the efficacy differences. Aside from the potential addition of dexamethasone, the dosing of carfilzomib could be optimized. Since the completion of this trial, the CHAMPION and ARROW trials have established the weekly higher dose of carfilzomib as the likely optimal method of carfilzomib dosing.^25^, ^26^ Thus, future studies exploring the combination of panobinostat and carfilzomib should consider this dosing strategy.

The majority of patients on this trial were carfilzomib‐naïve and thus the efficacy of this combination in carfilzomib‐exposed or refractory patient is unknown. This could limit the utility of this combination especially considering recent data from the CANDOR and IKEMA trials with carfilzomib and anti‐CD38 antibody combinations.^27^, ^28^ However, as more novel agents, including anti‐CD38 antibodies, get incorporated into first‐line and second‐line treatments, the combination of panobinostat and carfilzomib could be an attractive option for a patient with previous exposure to anti‐CD38 therapy who is being considered for carfilzomib‐based therapy in the relapsed setting. This was a small, non‐comparative trial, so we cannot draw direct conclusions about whether this regimen is more effective or has an improved safety profile than other panobinostat and carfilzomib schemas, or single‐agent carfilzomib. Furthermore, as per the nature of the rapidly evolving MM treatment landscape, standards of care have changed since the initiation of this trial. For example, many current real‐world patients are now relapsed or refractory to daratumumab. Nonetheless, the efficacy outcomes presented here for high‐risk, PI and IMiD refractory patients are promising, and further investigations are warranted. It would be of interest to investigate whether triplet or quadruplet regimens, with the addition of dexamethasone and/or lenalidomide, improve the benefit–risk profile. In addition, panobinostat in combination with ixazomib (another second‐generation PI) as an oral regimen for elderly patients who prefer not going into hospital, would be a useful regimen to investigate further. Early studies have produced limited data but nothing conclusive.^29^

## 5 CONCLUSION

The combination of panobinostat and carfilzomib is an effective steroid‐sparing regimen with a reasonable safety profile in this relapsed/refractory population. Further evaluation of this combination and as a backbone to triplet/quadruplet therapies is warranted.

## FINANCIAL DISCLOSURES

J.G.B.: reports research funding from AbbVie, Amgen, Acetylon, Bluebird, BMS, Celgene, Celularity, Constellation, CRISP Therapeutics, CURIS, EMD Sorono, Genentech, Glenmark, Janssen, Kesios, Lilly, Novartis, Poseida, Sanofi, Takeda, Teva, and Vivolux; consultancy for Amgen, BioClinica, Bluebird, BMS, Celgene, CRISPR Therapeutics, Janssen, Karyopharm, Kite Pharma, Legend, Prothena, SecuraBio, Servier, and Takeda.

T.G.: reports honoraria from Novartis.

E.F.: reports consultancy and honoraria from Amgen, Astra Zeneca, Cardinal Health, Celgene, GlaxoSmithKline, Janssen, Juno, Kite, and Takeda.

L.L.H.: reports honoraria and research funding from Onyx and Novartis.

J.R.M.: nothing to declare.

E.A.: nothing to declare.

I.W.F.: reports research funding from Agios, ArQule, Beigene, Calithera, Celgene, Constellation, Curis, Forma, Forty Seven, Genentech, Gilead, Incyte, Infinity, Janssen, Kite Pharma, Merck Novartis, Pfizer, Pharmacyclics, Portola, Roche, Seattle Genetics, Takeda, Teva, TG Therapeutics, Trillium, Verastem.

J.M.: reports consultancy for, honoraria from, and speakers bureau for Celgene, a Bristol‐Myers Squibb company; participation in a speakers' bureau for Seattle Genetics, Inc and Millennium Pharmaceuticals, Inc, a wholly owned subsidiary of Takeda Pharmaceuticals Limited.

## AUTHOR CONTRIBUTIONS

All authors contributed to patient enrolment and data collection; interpretation of data; manuscript review, and approval.

## Supporting information


**TABLE S1** Dose levels 5 and 6
**Table S2** Treatment received‐dose modification reasons
**Table S3** Cardiac toxicities^a^, regardless of causality (safety analyses).

## Data Availability

All data requests should be submitted to the corresponding author for consideration. Access to anonymized data may be granted following review.
