# Supplementary material for: A phase I/II study of the combination of panobinostat and carfilzomib in patients with relapsed or relapsed/refractory multiple myeloma: Final analysis of second dose‐expansion cohort
Source: Am J Hematol. 2021 Jan 28;96(4):428–35. doi: 10.1002/ajh.26088 (PMC7986798; doi:10.1002/ajh.26088)
Supplement: Supplementary file 1 — TABLE S1 Dose levels 5 and 6 Table S2 Treatment received‐dose modification reasons Table S3 Cardiac toxicitiesa, regardless of causality (safety analyses). [file AJH-96-428-s001.docx]

**SUPPLEMENTAL TABLE 1** Dose levels 5 and 6

| **28-day cycles** | **Cycle 1** | **Cycle 2 to progression** |
| --- | --- | --- |
| **Dose level 5** | | |
| Carfilzomib | 20 mg/m^2^ IV day 1, 2  56 mg/m^2^ IV day 8, 9, 15, 16 | 56 mg/m^2^ IV day 1, 2, 8, 9, 15, 16 |
| Panobinostat | 30 mg day PO 1, 3, 5, 15, 17, 19 | 30 mg day PO 1, 3, 5, 15, 17, 19 |
| **Dose level 6** | | |
| Carfilzomib | 20 mg/m^2^ IV day 1, 2  56 mg/m^2^ IV day 8, 9, 15, 16 | 56 mg/m^2^ IV day 1, 2, 8, 9, 15, 16 |
| Panobinostat | 20 mg day PO 1, 3, 5, 15, 17, 19 | 20 mg day PO 1, 3, 5, 15, 17, 19 |

Abbreviations: IV, intravenous; PO, orally.

**SUPPLEMENTAL TABLE 2** Treatment received-dose modification reasons

|  | **Dose level 5  (N = 3)** | **Dose level 6 (N = 32)** | **Total patients  (N = 35)** |
| --- | --- | --- | --- |
| **Number of patients (%)^a^** | | | |
| **Panobinostat-dose reductions reasons^b^** | | | |
| Hematological toxicity | 1 (33.3) | 12 (37.5) | 13 (37.1) |
| Thrombocytopenia | 1 (33.3) | 10 (31.2) | 11 (31.4) |
| Neutropenia | 0 | 2 (6.3) | 2 (5.7) |
| Non-hematological toxicity | 1 (33.3) | 15 (46.9) | 16 (45.7) |
| **Carfilzomib: dose reduction reasons^c^** | | | |
| **Hematological toxicity** | 1 (33.3) | 6 (18.7) | 7 (20.0) |
| Thrombocytopenia | 1 (33.3) | 6 (18.7) | 7 (20.0) |
| **Non-hematological toxicity** | 1(33.3) | 9 (28.1) | 10 (28.6) |
| **Panobinostat-dose interruption reasons^d^** | | | |
| **Hematological toxicity** | 0 | 6 (18.7) | 6 (17.1) |
| Thrombocytopenia | 0 | 5 (15.6) | 5 (14.3) |
| Anemia | 0 | 1 (3.1) | 1 (2.8) |
| **Non-hematological toxicity** | 2 (66.7) | 11 (34.4) | 13 (37.1) |
| **Carfilzomib-dose interruption reasons^e^** | | | |
| **Hematological toxicity** | 2 | 14 (43.8) | 16 (45.7) |
| Thrombocytopenia | 2 | 13 (40.6) | 15 (42.8) |
| Anemia | 0 | 2 (6.2) | 2 (5.7) |
| Neutropenia | 0 | 2 (6.2) | 2 (5.7) |
| **Non-hematological toxicity** | 2 | 23 (71.9) | 25 (71.4) |

**^a^**Please note that 1 dose modification may have been due to multiple reasons per patient; counts of patients are based on number of unique events per patient (not all occurrences per patient).
**^b^**Panobinostat dose was reduced due to thrombocytopenia in 10 (31.2%) of patients in DL6. 15 patients in dose level 6 had panobinostat dose reduction due to non-haematological reasons. Among these reasons, fatigue was the reason for panobinostat reduction in 3 (9.4%) patients.
**^c^**15 patients in DL6 had carfilzomib dose reduction due to hematological or non-hematological events. Of non-hematological events, the most frequent events were dyspnea: 2 (6.2%) and fatigue: 2 (6.2%).
**^d^**20 (62.5%) of patients in DL6 had panobinostat dose interruptions due to hematological, non-hematological events or other non-adverse event reasons. 6 (18.7%) patients had hematological events as the reason for dose interruption. Thrombocytopenia was the reason for panobinostat interruptions in 5 (15.6%) patients. 11 patients had at least 1 panobinostat dose interruption due to non-hematolgocal reasons. Among these reasons, pneumonia was the reason in 3 (9.3%) patients, hypokalemia, hypomagnesemia, cough and fever were reasons for panobinostat interruptions in 2 (6.2%) patients per each reason. Panobinostat was discontinued in 3 patients of dose level 6 and 1 patient in dose level 5 due to thrombocytopenia.
**^e^**28 patients in DL6 had 1 more carfilzomib interruption due to hematological, non-hematological and/or other reasons. Among hematological toxicities, thrombocytopenia was the reason for drug interruption in 13 (40.6%) patients. 23 patients in dose level 6 had at least 1 drug interruptions due to non-hematological events; among all non-hematological events, fever was the reason for dose interruption in 5 (15.6%) and nausea was the reason for dose interruption in 4 (12.5%) patients.

**SUPPLEMENTAL TABLE 3** Cardiac toxicities^a^, regardless of causality (safety analyses)

| AEs, n (%) | Grade 1 | Grade 2 | Grade 3 | Grade 4 | Grade 5 | Total (N = 33) |
| --- | --- | --- | --- | --- | --- | --- |
| Atrial fibrillation | 0 | 0 | 0 | 1 (3.0) | 0 | 1 (3.0) |
| Bradycardia | 1 (3.0) | 0 | 0 | 0 | 0 | 1 (3.0) |
| Cardiac chest pain | 0 | 1 (3.0) | 0 | 0 | 0 | 1 (3.0) |
| Cardiac flutter | 1 (3.0) | 0 | 0 | 0 | 0 | 1 (3.0) |
| Heart failure | 0 | 0 | 1 (3.0) | 0 | 0 | 1 (3.0) |
| Palpitations | 1 (3.0) | 0 | 0 | 0 | 0 | 1 (3.0) |
| Premature atrial contractions | 1 (3.0) | 0 | 0 | 0 | 0 | 1 (3.0) |
| Premature ventricular contractions | 1 (3.0) | 0 | 0 | 0 | 0 | 1 (3.0) |
| Tachycardia | 1 (3.0) | 0 | 0 | 0 | 0 | 1 (3.0) |
| Dyspnea | 5 (15.2) | 4 (12.1) | 3 (9.1) | 1 (3.0) | 0 | 13 (39.4) |
| Hypertension | 1 (3.0) | 2 (6.1) | 3 (9.1) | 0 | 0 | 6 (18.2) |
| Any cardiac toxicity^b^ | 5 (15.2) | 6 (18.2) | 6 (18.2) | 1 (3.0) | 0 | 18 (54.5) |

Abbreviation: AEs, adverse events.
Percentages are shown for patients in dose level 6; no patients in dose level 5 group experienced cardiac toxicities.
^a^No patients in dose levels 5 and 6 experienced prolonged QTc.
^b^18 patients experienced cardiac toxicities. Percentages in this row are shown based on the highest grade that each patient encountered.
